# Marrow-Infiltrating Lymphocytes – Role in Biology and Cancer Therapy

**DOI:** 10.3389/fimmu.2016.00112

**Published:** 2016-03-30

**Authors:** Ivan Borrello, Kimberly A. Noonan

**Affiliations:** ^1^Department of Oncology, Johns Hopkins University, Baltimore, MD, USA

**Keywords:** marrow-infiltrating lymphocytes, bone marrow, cancer immunotherapy

## Abstract

The past several years have witnessed the acceptance of immunotherapy into the mainstream of therapies for patients with cancer. This has been driven by the clinical successes of antibodies to the checkpoint inhibitors, CTLA-4 and PD-1, capable of imparting long-term remissions in several solid tumors as well as Hodgkin’s lymphoma (1) and the therapeutic successes of adoptive T-cell transfer with chimeric antigen receptors (2) or modified T-cell receptors (3) that have mostly utilized peripheral T-cells. One emerging area of therapeutic T cell intervention has been the utilization of marrow-infiltrating lymphocytes (MILs) – a novel form of adoptive T-cell therapy. This approach was initially developed to increase the likelihood of a precursor T-cell population with an enhanced tumor specificity in bone marrow (BM)-derived malignancies. However, the unique attributes of BM T-cells and their interaction with their microenvironment provide significant rationale to utilize these cells therapeutically in diseases that extend beyond hematologic malignancies.

## Introduction

Most adoptive T-cell therapy has utilized the circulating pool of peripheral blood lymphocytes (PBLs). This is likely due to the relative ease of obtaining these cells through large volume pheresis. An alternative approach pioneered by Dr. Rosenberg and colleagues at the National Cancer Institute has focused on the use of tumor infiltrating lymphocytes (TILs) in patients with metastatic melanoma (4). The rationale for TILs is that tumor-specific T-cells are more likely to be found in proximity of the tumor. This has proven to be true, and several clinical studies have demonstrated appreciable tumor specificity with measurable antitumor efficacy. However, limitations exist with the TIL approach. First, in contrast to PBLs, TILs are not present in all individuals, they require a surgical procedure for harvesting, and the expansion protocols are often both lengthy and costly.

In hematologic malignancies, MILs likely offer the same benefits of TILs. Being obtained from the tumor microenvironment in myeloma as well as several other hematologic malignancies, these cells demonstrate heightened tumor specificity upon activation and expansion (5). However, the unique immunologic properties of bone marrow (BM) impart essential properties that could possibly make them an even better source of T-cells for adoptive therapy approaches than their PBL counterparts.

## Rationale for Use of MILs in Adoptive T Cell Therapy

Effective adoptive T-cell therapy requires T-cells to possess important attributes. At a minimum, they must (1) possess endogenous tumor specificity, (2) be capable of trafficking to the tumor site upon infusion, (3) kill the tumor upon encounter, and (4) persist over time. MILs possess these properties in large part because of the unique immune environment present within the BM.

T-cells compose only 3–8% of the population within the BM (6). However, the BM plays a critical role in priming naive T-cells (7), serving as a reservoir of antigen experienced CD8 memory T cells (8), and the site of homeostatic proliferation of both CD4 and CD8 T-cells (9, 10). While the BM lacks the organized structure of the lymph node or spleen, it does provide an environment that supports appropriate T-cell development in the absence of the thymus (11) and is capable of forming lymphoid follicles which increase with inflammatory, autoimmune, or infectious states (12). Despite this immune function, the BM lacks lymphatic vessels and is vascularized only by blood vessels. The absence of lymphatic vessels likely enables the massive lymphocyte recirculation that occurs daily. However, entrance of central memory T_CM_ cells into the BM is a more tightly regulated process mediated by an interaction between L-, P-, and E-selectins, whereas arrest within the BM requires the VCAM-1/α4β1 pathway (13).

**Figure 1:**
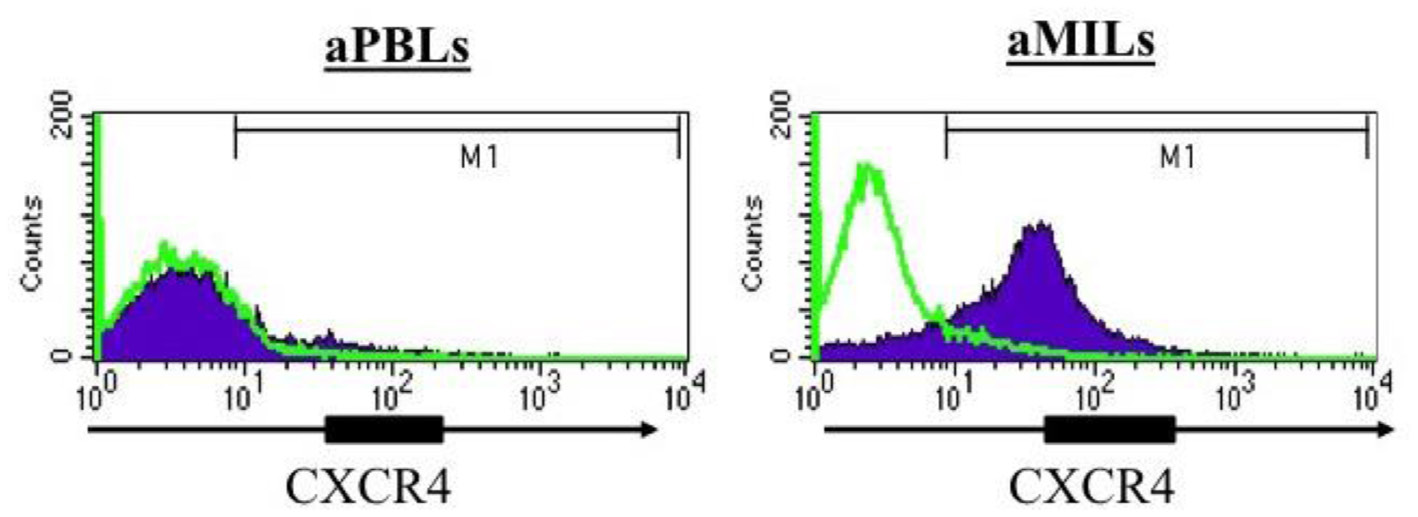
**CXCR4 expression in activated PBLs compared to activated MILs**. The T cells were activated with anti-CD3/CD28 beads, and CXCR4 expression was then assessed by flow cytometry.

Stromal-derived factor-1/CXCL12 is highly expressed in the BM, and its cognate receptor, CXCR4, is upregulated on memory T cells by IL-15 and increases adhesion of T_CM_ to the BM microvessels (13, 14). Furthermore, memory T-cells have higher expression of CXCR4 compared to naive T-cells as its expression appears to be affected by the presence of antigen and various cytokines (14). In myeloma, we have confirmed the expression of CXCR4 on MILs, whereas no detectable expression was appreciated on PBLs from the same patients (Figure 1) (5). Of note, expression was even greater following activation with anti-CD3/CD28 beads. In hematologic malignancies, this expression may provide important advantages to MILs, such as maximizing trafficking of the T-cells to the tumor microenvironment and thus further increase their therapeutic benefit in adoptive T-cell therapy. In addition to the possibility that CXCR4 expression on MILs can increase their trafficking to the BM, its increased expression on memory T-cells also provides another justification for the use of MILs in adoptive T-cell therapy. Specifically, CXCR4 is also involved in promotion of CD8 T_CM_ homeostatic proliferation and maintenance (15). CD8 T_CM_ imparts long-term memory and are essential to maximizing the overall efficacy of adoptive T-cell therapy (16). The combined increased expression of CXCR4 on memory T-cells, the increased trafficking of these T-cells in response to CXCL12 to the BM, and the subsequent enrichment of memory T cells within the BM all underscore the unique attributes of BM resident T-cells that make them an ideal source for adoptive T-cell therapeutic approaches. Trafficking to the tumor site, BM, and persistence over time are two important properties for T-cells in the treatment of hematologic malignancies.

## Role of BM T Cells in Disease Regulation and Systemic Immunity

It is logical to assume that T-cells within a certain microenvironment most likely possess an enhanced antigenic specificity for the cells residing within that organ. This is certainly the rationale behind the therapeutic use of TILs for the treatment of metastatic melanoma and other diseases, in which they have been employed and was the primary assumption used in the clinical development of MILs in multiple myeloma by our group. However, as outlined above, the BM represents a unique organ with several important immune attributes, which can be employed for therapeutic purposes but can also contribute to the pathology of disease.

### Hematologic Malignancies

The BM has been described as a reservoir for Tregs (17). Interestingly, this observation was made in healthy hosts, and our findings in myeloma demonstrated the opposite – more Tregs present in the peripheral blood than BM (18). This paradox was ultimately resolved by our demonstration that the large amounts of IL-6 produced by the myelomatous plasma cells in combination with TGF-β (as well as IL-1β and IL-21) were responsible for the skewing of CD4 away from a Treg phenotype toward a Th17 phenotype that was responsible for the activation of osteoclasts and the development of lytic bone lesions (18) as well as the promotion of myeloma cell growth (19). Underscoring the importance of the immune composition of the BM, it was the extent of the Th17 phenotype of the BM (but not blood) T-cells and not the myeloma cell concentration that correlated to the extent of bone disease (18). Interestingly, activation of MILs with anti-CD3/CD28 resulted in a significant skewing toward a Th1 phenotype and the addition of activated MILs to an osteoclast differentiation assay significantly reduced the outgrowth of osteoclasts (18). Taken together, these data not only provide an explanation for the immune-mediated contribution to lytic bone disease in myeloma but also suggest that an adoptive T cell approach, such as the utilization of Th1-skewed-activated MILs, could possibly also exert a benefit in terms of reducing bone disease.

### Viral Responses

The role of the BM in serving as a reservoir of antigen-experienced T-cells is likely also mediated by the ability of the BM to effectively present antigen. In fact, it has been demonstrated that differentiated dendritic cells (DCs) traffic and home to the BM where they then form stable antigen-dependent interactions with T_CM_ (20). This interaction is likely one of the key mediators responsible for the maintenance of central memory in the BM. It is this attribute that also enables the BM to enrich for viral-specific memory T-cells. In a murine LCMV model, it was shown that no significant differences existed between the precursor frequency of LCMV-specific T-cells in the spleen and BM during the acute infection. Furthermore, long-term immunity was found in the BM and the spleen, and the adoptive transfer of BM from LCMV-infected mice was able to effectively protect against viral infection (21). Chronic viral infections, such as EBV, also demonstrate an increase in BM-specific T-cells to lytic antigens compared to the blood, which was independent of viral load (22). More importantly, that study showed a unique expression of homing receptors present on T cells obtained from the BM and not observed on T cells from other compartments.

### Solid Tumors

In contrast to the two abovementioned conditions, in solid tumors (especially in the non-metastatic setting), it is unlikely that the BM will contain clinically detectable amounts of tumor. In breast cancer, tumor-specific T-cells have been identified in the BM to a greater extent than in the blood even in conditions in which tumor could not be detected by nested PCR (23). Similar results were also observed in melanoma and pancreatic cancer; although in the former, BM T-cell recognition was more commonly seen in patients with metastatic disease (24, 25). Based on these results, studies are currently ongoing examining the ability to utilize MILs as a therapeutic source of T cells in various solid tumors.

## Therapeutic Role of MILs

The abovementioned attributes underscore the uniqueness of the MILs as more than simply the TILs of hematologic malignancies. In fact, the demonstration by various groups that antigen-specific cells can be found even in the absence of known tumor or viral involvement of the BM provides the rationale for their therapeutic use in those disease settings as well.

### Multiple Myeloma

**Figure 2:**
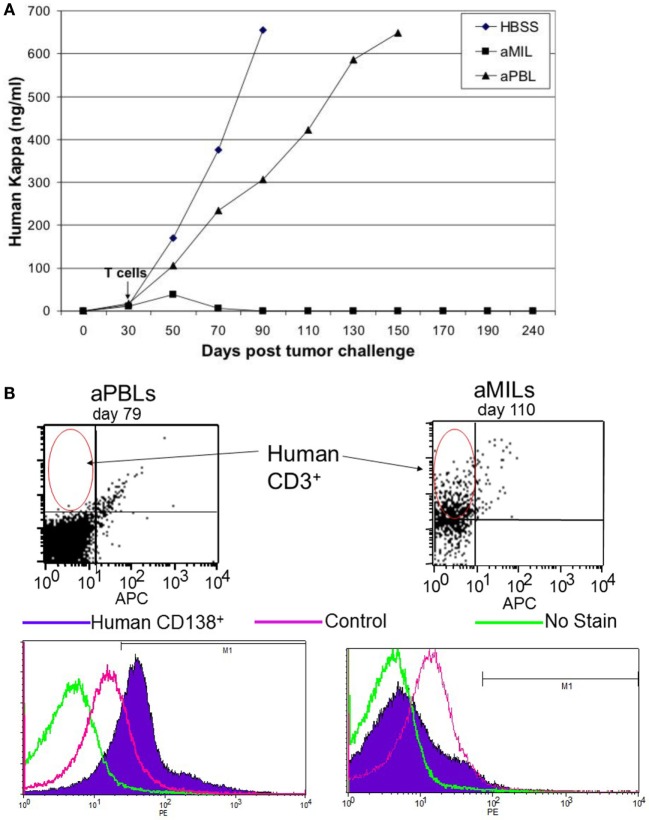
**Activated MILs exert a potent antitumor MILs**. **(A)** NOD/SCID mice (10 mice per group) were challenged with the H929 myeloma cell line and followed until a detectable kappa light chain was present in the blood consistent with engraftment. The mice were challenged with either activated PBLs or activated MILs and followed for overall survival. **(B)** To determine the ability of these T cells to traffic and exert antimyeloma activity, the mice were sacrificed at the indicated time points. When mice that received aPBL died or conversely a mouse that received aMILs was sacked on D110 to analyze the BM was stained for either human CD3 cells or CD138^+^ myeloma. As shown, the mice that received MILs showed significant T cell trafficking to the BM and no detectable myeloma.

Marrow-infiltrating lymphocytes do appear to possess many of the essential features that make them the ideal for adoptive T cell therapy and more specifically, for hematologic malignancies. They have an endogenous antigenic specificity that is broad and targets antigens present on both the mature myelomatous plasma cells as well as their clonogenic precursors. In NOD/SCID models, MILs have shown the ability to traffic to the BM, to persist with an activated phenotype, and to exert measurable antitumor activity (Figure 2). Our clinical data demonstrate a direct correlation between the ability to achieve a complete remission (CR), following the adoptive transfer of activated MILs and the presence of significant tumor-specific immunity to myeloma cell lysate (26). Studies to specifically determine the specific antigenic recognition are ongoing. However, the antigen-specific immunity appears to be both CD4- and CD8 mediated.

These initial studies have led to the development of clinical trials for myeloma in the autologous transplant setting. This involved the bedside harvest of 200 ml of BM, which was well tolerated with conscious sedation and from which MILs were then expanded in all patients. Of note, MILs were not isolated prior to activation but rather were expanded within the BM microenvironment because the presence of antigen during the T-cell expansion was shown to be critical to maintaining the tumor specificity of the MILs while subjected to the potent polyclonal expansion of CD3/CD28 stimulation. However, underscoring the unique attribute of MILs, we have demonstrated that it is more than the presence of antigen during the expansion that is responsible for the antigenic specificity as PBLs expanded within the BM environment showed only a slight increase in the tumor specificity but significantly less could be achieved with MILs (5). In our clinical study, we observed an increase in the tumor specificity of the MILs in all patients. However, the magnitude of this specificity varied significantly post-expansion. Interestingly, a direct correlation existed between the extent of the tumor specificity obtained *ex vivo* and the clinical outcomes of the patients – higher tumor specificity and increased production of both IL-2 and IFNγ of the *ex vivo* product was associated with CRs (26). Several properties of MILs at baseline also seemed to correlate to clinical outcomes. Specifically, patients whose pre-expansion MILs had significant percentage of CD8 T_CM_ and low spontaneous IFNγ production were more likely to achieve a CR to therapy. The demonstration of a direct correlation between the immune parameters of the T-cell product and clinical outcomes speaks to the endogenous tumor specificity of MILs.

Marrow-infiltrating lymphocytes were successfully administered without the development of a cytokine release syndrome or significant lymphocytosis. This differs significantly from the infusion of activated PBL which, even when unmodified, demonstrated a significant lymphocytosis *in vivo*. The absence of these effects, following the infusion of MILs, could be related to the infusion of a cell dose that was below the threshold for expansion or could reflect a different trafficking pattern of MILs. However, we did observe the development of a rash consistent with an acute autologous graft-vs.-host disease in roughly 20% of patients. This was mostly limited to the skin and resolved spontaneously. No long-term sequelae were evident in patients who received MILs.

A major objective of these studies was to determine the extent of immune responsiveness within the tumor microenvironment. For this reason, the immune monitoring was only performed on BM samples obtained at time points pre- and posttransplant. In addition to the direct correlation observed between the *ex vivo* expanded product and clinical outcomes, the MILs obtained from the patients up to 1 year posttransplant also demonstrated a direct correlation between the tumor specificity and the clinical response to therapy. MILs with the greatest antimyeloma specificity were observed in patients who achieved a CR. The CR patients also demonstrated an increase in CD8 cytotoxic activity as measured by both granzyme B and perforin production, an increase in IFNγ production, and a decrease in IL-6 production. One obstacle for many non-gene-modified T-cell studies has been the absence of persistence of *ex vivo* activated T-cells. We attempted to determine this in our study by vaccinating our patients the polyvalent pneumococcal vaccine (PCV) prior to harvesting the MILs and then reinfusing the MILs into a host with a T-cell-depleted graft where the MILs constituted more than 95% of the adoptively transferred T cells. As such, it is fair to assume that the vast majority of PCV-specific MILs were those adoptive transferred. Our ability to detect PCV-specific MILs up to 1 year posttransplant, especially in patients who achieved a CR strongly, suggests their persistence.

Ongoing clinical studies are addressing approaches aimed at increasing the efficacy of MILs adoptive cell therapy. These have included the addition of a cellular allogeneic myeloma vaccine (myeloma-GVAX) as well as the use of the lenalidomide following immune reconstitution. The former attempts to increase the precursor frequency of myeloma-specific MILs, and the latter attempts to maintain the activation state of the MILs.

### Post-Allogeneic Transplant MILs

A major unmet need in allogeneic stem cell transplantation remains the difficulty of dealing with relapse disease following the transplant. The current standard approach is the use of donor lymphocyte infusions (DLI), which has a limited efficacy but is associated with a significant incidence of graft-vs.-host disease (GVHD) that can be in excess of 50% (27). The recent development at our center are allogeneic transplants with the use of posttransplant cyclophosphamide (PTCy), which has significantly reduced the incidence of GVHD and has enabled transplants to be performed across HLA barriers without significant increases in the morbidity or mortality compared to HLA-identical transplants (28). Because of the PTCy elimination of alloreactive T-cells, we hypothesized that MILs obtained from these patients posttransplant could be utilized therapeutically as a modified version of DLI with a better toxicity profile and heightened tumor specificity. The rationale is that the PTCy would have eliminated the alloreactive T-cells and the BM would possess the donor-derived tumor-specific T-cells. As such, we should be able to obtain MILs from these patients in the post-allogeneic transplant setting, activate, and expand these cells. The expectation is that the MILs would have heightened tumor specificity, effectively traffic to the BM, eradicate existing disease, and cause minimal, if any, GVHD. In preclinical experiments, the tumor specificity of MILs, following anti-CD3/CD28 activation, was observed in all patients examined, and one patient in whom a tetramer existed for the HLA-A2^+^ PR-1 peptide, the precursor frequency of MILs went from 1 to 17.8% following expansion (unpublished data). Considering the polyclonal expansion achieved with anti-CD3/CD28 stimulation, the theoretical ability to increase the antigenic frequency to numerous antigens by this percentage with minimal associated toxicity offers significant promise as an approach that should significantly improve the therapeutic outcomes for patients relapsing following an allogeneic BMT.

## Solid Tumors

The positive prognostic value in terms of overall survival of TILs in many solid tumors points to a clear role of the immune system in regulating cancer growth (29–31). This has been further evidenced by the clinical benefits observed in many metastatic melanoma patients treated with TILs (32). However, a major limitation to such an approach is that not all patients have TILs that can be harvested, and the harvesting is a surgical procedure with all its limitations.

The presence of tumor-specific T-cells in the BM of patients with solid tumors opens up the prospect of utilizing MILs for therapeutic adoptive T-cell therapy in this setting, despite the presence of tumor within the BM. This approach has been examined in patients with metastatic breast cancer (33). In that study, 16 HLA-A2^+^ patients had MILs cocultured with autologous DCs and an allogeneic breast cancer cell line, MCF7. An average of 2 × 10^7^ T-cells were infused without a lymphoablative preparative regimen. There was a direct correlation between the number of tumor-specific infused T-cells, the presence of a memory phenotype, and the absence of a Th2 phenotype with the overall response. Interestingly, patients with evidence of tumor-specific immunity in PBL showed a significantly longer median survival compared to non-responders (58.6 vs. 13.6 months; *p* = 0.009) (34).

## Chimeric Antigen Receptor-Modified T Cells

Chimeric antigen receptor (CAR) T-cells have demonstrated the ability to eradicate significant tumor burdens in patients with chronic lymphocytic leukemia (35) and acute lymphoblastic leukemia (36, 37). While the response rates are impressively around 70–90% in ALL with a 6-month event-free survival of 67%, the overall mechanisms of relapse are not completely understood. The development of antigen-escape variants are clearly observed in a fraction of the patients (38). One approach being developed to overcome this problem is to utilize an additional CAR, which in the case of ALL has been developed to target CD22 (39). An alternative approach could be to utilize a cell with an intrinsically broader antigenic specificity than that found on a PBL. MILs would appear to possess such properties and as such could potentially serve as a better source of T-cells for CAR-based adoptive T-cell therapy.

## Conclusion

The BM represents a unique immunologic environment that serves both as a reservoir of memory T-cells and can effectively prime naive T-cells. This is, in large part, due to the efficient processing and presentation of antigens by resident APCs. The major benefit of this biology is that MILs possess many essential properties that make them ideal for adoptive T-cell therapy: they are enriched for T_CM_, they possess a broad antigenic specificity, effectively traffic to the BM upon reinfusion, and are capable of significant cytotoxicity. Furthermore, the presence of antigen-specific T-cells in the BM in patients with certain viral infections, as well as solid tumors, potentially provides the rationale to utilize MILs for adoptive T-cell therapy.

## Author Contributions

IB and KN contributed to the design of experiments, analysis, and writing of the manuscript.

## Conflict of Interest Statement

The authors declare that the research was conducted in the absence of any commercial or financial relationships that could be construed as a potential conflict of interest.

## References

[1] Pardoll DM. The blockade of immune checkpoints in cancer immunotherapy. Nat Rev Cancer (2012) 12(4):252–64. 10.1038/nrc3239 22437870 PMC4856023

[2] Sadelain M. CAR therapy: the CD19 paradigm. J Clin Invest (2015) 125(9):3392–400. 10.1172/JCI80010 26325036 PMC4588281

[3] Bonini C Mondino A. Adoptive T-cell therapy for cancer: the era of engineered T cells. Eur J Immunol (2015) 45(9):2457–69. 10.1002/eji.201545552 26202766

[4] Dudley ME Wunderlich JR Robbins PF Yang JC Hwu P Schwartzentruber DJ Cancer regression and autoimmunity in patients after clonal repopulation with antitumor lymphocytes. Science (2002) 298(5594):850–4. 10.1126/science.1076514 12242449 PMC1764179

[5] Noonan K Matsui W Serafini P Carbley R Tan G Khalili J Activated marrow-infiltrating lymphocytes effectively target plasma cells and their clonogenic precursors. Cancer Res (2005) 65(5):2026–34. 10.1158/0008-5472.CAN-04-3337 15753403

[6] Schirrmacher V Feuerer M Fournier P Ahlert T Umansky V Beckhove P T-cell priming in bone marrow: the potential for long-lasting protective anti-tumor immunity. Trends Mol Med (2003) 9(12):526–34. 10.1016/j.molmed.2003.10.001 14659467

[7] Feuerer M Beckhove P Garbi N Mahnke Y Limmer A Hommel M Bone marrow as a priming site for T-cell responses to blood-borne antigen. Nat Med (2003) 9(9):1151–7. 10.1038/nm914 12910264

[8] Feuerer M Beckhove P Bai L Solomayer EF Bastert G Diel IJ Therapy of human tumors in NOD/SCID mice with patient-derived reactivated memory T cells from bone marrow. Nat Med (2001) 7(4):452–8. 10.1038/86523 11283672

[9] Di Rosa F Santoni A. Bone marrow CD8 T cells are in a different activation state than those in lymphoid periphery. Eur J Immunol (2002) 32(7):1873–80. 10.1002/1521-4141(200207)32:7<1873::AID-IMMU1873>3.0.CO;2-P 12115606

[10] Parretta E Cassese G Santoni A Guardiola J Vecchio A Di Rosa F. Kinetics of in vivo proliferation and death of memory and naive CD8 T cells: parameter estimation based on 5-bromo-2’-deoxyuridine incorporation in spleen, lymph nodes, and bone marrow. J Immunol (2008) 180(11):7230–9. 10.4049/jimmunol.180.11.7230 18490722

[11] Dejbakhsh-Jones S Jerabek L Weissman IL Strober S. Extrathymic maturation of alpha beta T cells from hemopoietic stem cells. J Immunol (1995) 155(7):3338–44. 7561027

[12] Zhao E Xu H Wang L Kryczek I Wu K Hu Y Bone marrow and the control of immunity. Cell Mol Immunol (2012) 9(1):11–9. 10.1038/cmi.2011.47 22020068 PMC3251706

[13] Mazo IB Honczarenko M Leung H Cavanagh LL Bonasio R Weninger W Bone marrow is a major reservoir and site of recruitment for central memory CD8+ T cells. Immunity (2005) 22(2):259–70. 10.1016/j.immuni.2005.01.008 15723813

[14] Jourdan P Vendrell JP Huguet MF Segondy M Bousquet J Pene J Cytokines and cell surface molecules independently induce CXCR4 expression on CD4+ CCR7+ human memory T cells. J Immunol (2000) 165(2):716–24. 10.4049/jimmunol.165.2.716 10878344

[15] Chaix J Nish SA Lin WH Rothman NJ Ding L Wherry EJ Cutting edge: CXCR4 is critical for CD8+ memory T cell homeostatic self-renewal but not rechallenge self-renewal. J Immunol (2014) 193(3):1013–6. 10.4049/jimmunol.1400488 24973450 PMC4108510

[16] Berger C Jensen MC Lansdorp PM Gough M Elliott C Riddell SR. Adoptive transfer of effector CD8+ T cells derived from central memory cells establishes persistent T cell memory in primates. J Clin Invest (2008) 118(1):294–305. 10.1172/JCI32103 18060041 PMC2104476

[17] Zou L Barnett B Safah H Larussa VF Evdemon-Hogan M Mottram P Bone marrow is a reservoir for CD4+CD25+ regulatory T cells that traffic through CXCL12/CXCR4 signals. Cancer Res (2004) 64(22):8451–5. 10.1158/0008-5472.CAN-04-1987 15548717

[18] Noonan K Marchionni L Anderson J Pardoll D Roodman GD Borrello I A novel role of IL-17-producing lymphocytes in mediating lytic bone disease in multiple myeloma. Blood (2010) 116(18):3554–63. 10.1182/blood-2010-05-283895 20664052 PMC4017298

[19] Prabhala RH Pelluru D Fulciniti M Prabhala HK Nanjappa P Song W Elevated IL-17 produced by TH17 cells promotes myeloma cell growth and inhibits immune function in multiple myeloma. Blood (2010) 115(26):5385–92. 10.1182/blood-2009-10-246660 20395418 PMC2902136

[20] Cavanagh LL Bonasio R Mazo IB Halin C Cheng G van der Velden AW Activation of bone marrow-resident memory T cells by circulating, antigen-bearing dendritic cells. Nat Immunol (2005) 6(10):1029–37. 10.1038/ni1249 16155571 PMC1780273

[21] Slifka MK Whitmire JK Ahmed R. Bone marrow contains virus-specific cytotoxic T lymphocytes. Blood (1997) 90(5):2103–8. 9292550

[22] Palendira U Chinn R Raza W Piper K Pratt G Machado L Selective accumulation of virus-specific CD8+ T cells with unique homing phenotype within the human bone marrow. Blood (2008) 112(8):3293–302. 10.1182/blood-2008-02-138040 18635810

[23] Feuerer M Rocha M Bai L Umansky V Solomayer EF Bastert G Enrichment of memory T cells and other profound immunological changes in the bone marrow from untreated breast cancer patients. Int J Cancer (2001) 92(1):96–105. 10.1002/1097-0215(200102)9999:9999<::AID-IJC1152>3.0.CO;2-Q 11279612

[24] Muller-Berghaus J Ehlert K Ugurel S Umansky V Bucur M Schirrmacher V Melanoma-reactive T cells in the bone marrow of melanoma patients: association with disease stage and disease duration. Cancer Res (2006) 66(12):5997–6001. 10.1158/0008-5472.CAN-04-0484 16778169

[25] Schmitz-Winnenthal FH Volk C Z’Graggen K Galindo L Nummer D Ziouta Y High frequencies of functional tumor-reactive T cells in bone marrow and blood of pancreatic cancer patients. Cancer Res (2005) 65(21):10079–87. 10.1158/0008-5472.CAN-05-1098 16267034

[26] Noonan KA Huff CA Davis J Lemas MV Fiorino S Bitzan J Adoptive transfer of activated marrow-infiltrating lymphocytes induces measurable antitumor immunity in the bone marrow in multiple myeloma. Sci Transl Med (2015) 7(288):288ra78 10.1126/scitranslmed.aaa7014 PMC4634889 25995224

[27] Luznik L Fuchs EJ. Donor lymphocyte infusions to treat hematologic malignancies in relapse after allogeneic blood or marrow transplantation. Cancer Control (2002) 9(2):123–37. 10.1177/107327480200900205 11965233

[28] Luznik L Jones RJ Fuchs EJ. High-dose cyclophosphamide for graft-versus-host disease prevention. Curr Opin Hematol (2010) 17(6):493–9. 10.1097/MOH.0b013e32833eaf1b 20827187 PMC3138214

[29] Galon J Costes A Sanchez-Cabo F Kirilovsky A Mlecnik B Lagorce-Pages C Type, density, and location of immune cells within human colorectal tumors predict clinical outcome. Science (2006) 313(5795):1960–4. 10.1126/science.1129139 17008531

[30] Zhang L Yang N Conejo-Garcia JR Katsaros D Mohamed-Hadley A Fracchioli S Expression of endocrine gland-derived vascular endothelial growth factor in ovarian carcinoma. Clin Cancer Res (2003) 9(1):264–72. 12538479

[31] Salgado R Denkert C Campbell C Savas P Nucifero P Aura C Tumor-infiltrating lymphocytes and associations with pathological complete response and event-free survival in HER2-positive early-stage breast cancer treated with lapatinib and trastuzumab: a secondary analysis of the NeoALTTO Trial. JAMA Oncol (2015) 1(4):448–54. 10.1001/jamaoncol.2015.0830 26181252 PMC5551492

[32] Hinrichs CS Rosenberg SA. Exploiting the curative potential of adoptive T-cell therapy for cancer. Immunol Rev (2014) 257(1):56–71. 10.1111/imr.12132 24329789 PMC3920180

[33] Schuetz F Ehlert K Ge Y Schneeweiss A Rom J Inzkirweli N Treatment of advanced metastasized breast cancer with bone marrow-derived tumour-reactive memory T cells: a pilot clinical study. Cancer Immunol Immunother (2009) 58(6):887–900. 10.1007/s00262-008-0605-3 18998129 PMC11030204

[34] Domschke C Ge Y Bernhardt I Schott S Keim S Juenger S Long-term survival after adoptive bone marrow T cell therapy of advanced metastasized breast cancer: follow-up analysis of a clinical pilot trial. Cancer Immunol Immunother (2013) 62(6):1053–60. 10.1007/s00262-013-1414-x 23595207 PMC11029475

[35] Porter DL Levine BL Kalos M Bagg A June CH. Chimeric antigen receptor-modified T cells in chronic lymphoid leukemia. N Engl J Med (2011) 365(8):725–33. 10.1056/NEJMoa1103849 21830940 PMC3387277

[36] Maude SL Frey N Shaw PA Aplenc R Barrett DM Bunin NJ Chimeric antigen receptor T cells for sustained remissions in leukemia. N Engl J Med (2014) 371(16):1507–17. 10.1056/NEJMoa1407222 25317870 PMC4267531

[37] Davila ML Riviere I Wang X Bartido S Park J Curran K Efficacy and toxicity management of 19-28z CAR T cell therapy in B cell acute lymphoblastic leukemia. Sci Transl Med (2014) 6(224):224ra25 10.1126/scitranslmed.3008226 PMC4684949 24553386

[38] Maude SL Teachey DT Porter DL Grupp SA. CD19-targeted chimeric antigen receptor T-cell therapy for acute lymphoblastic leukemia. Blood (2015) 125(26):4017–23. 10.1182/blood-2014-12-580068 25999455 PMC4481592

[39] Haso W Lee DW Shah NN Stetler-Stevenson M Yuan CM Pastan IH Anti-CD22-chimeric antigen receptors targeting B-cell precursor acute lymphoblastic leukemia. Blood (2013) 121(7):1165–74. 10.1182/blood-2012-06-438002 23243285 PMC3575759

